# Electrochemical Gating of Tricarboxylic Acid Cycle in Electricity-Producing Bacterial Cells of *Shewanella*

**DOI:** 10.1371/journal.pone.0072901

**Published:** 2013-08-20

**Authors:** Shoichi Matsuda, Huan Liu, Atsushi Kouzuma, Kazuya Watanabe, Kazuhito Hashimoto, Shuji Nakanishi

**Affiliations:** 1 Department of Applied Chemistry, the University of Tokyo, Tokyo, Japan; 2 Research Center for Advanced Science and Technology, the University of Tokyo, Tokyo, Japan; 3 Exploratory Research for Advanced Technology/Japan Science and Technology Agency, HASHIMOTO Light Energy Conversion Project, Tokyo, Japan; 4 School of Life Sciences, Tokyo University of Pharmacy and Life Sciences, Hachioji, Tokyo, Japan; RMIT University, Australia

## Abstract

Energy-conversion systems mediated by bacterial metabolism have recently attracted much attention, and therefore, demands for tuning of bacterial metabolism are increasing. It is widely recognized that intracellular redox atmosphere which is generally tuned by dissolved oxygen concentration or by appropriate selection of an electron acceptor for respiration is one of the important factors determining the bacterial metabolism. In general, electrochemical approaches are valuable for regulation of redox-active objects. However, the intracellular redox conditions are extremely difficult to control electrochemically because of the presence of insulative phospholipid bilayer membranes. In the present work, the limitation can be overcome by use of the bacterial genus *Shewanella* , which consists of species that are able to respire via cytochromes abundantly expressed in their outer-membrane with solid-state electron acceptors, including anodes. The electrochemical characterization and the gene expression analysis revealed that the activity of tricarboxylic acid (TCA) cycle in *Shewanella* cells can be reversibly gated simply by changing the anode potential. Importantly, our present results for *Shewanella* cells cultured in an electrochemical system under poised potential conditions showed the opposite relationship between the current and electron acceptor energy level, and indicate that this unique behavior originates from deactivation of the TCA cycle in the (over-)oxidative region. Our result obtained in this study is the first demonstration of the electrochemical gating of TCA cycle of living cells. And we believe that our findings will contribute to a deeper understanding of redox-dependent regulation systems in living cells, in which the intracellular redox atmosphere is a critical factor determining the regulation of various metabolic and genetic processes.

## Introduction

The metabolism of living cells is composed of numerous redox reactions that generate energy for growth and cellular maintenance. It is widely recognized that the intracellular redox condition is a critical factor determining the regulation of various metabolic and genetic processes [1–3]. Among such redox-responsive regulation systems, redox-dependent gating of the tricarboxylic acid (TCA) cycle governed by the ArcB/ArcA two-component signal transduction system is one of the redox-active system that has been most extensively investigated to date [4–6]. Under aerobic (i.e., oxidative) conditions, the ArcB sensor kinase autophosphorylates and then transphosphorylates ArcA (a global transcriptional regulator), triggering up-regulation of TCA cycle-related genes. In contrast, the kinase activity of ArcB is directly inhibited under anaerobic (i.e., reductive) conditions, by quinone electron carriers, resulting in deactivation of the TCA cycle.

In general, electrochemical approaches are valuable for regulation of redox-active compounds. However, the intracellular redox conditions are extremely difficult to control electrochemically because of the presence of insulative phospholipid bilayer membranes. This limitation can be overcome by use of the bacterial genus *Shewanella* , which consists of species that are able to respire via cytochromes abundantly expressed in their outer-membrane (outer-membrane cytochromes, OMCs) with solid-state electron acceptors, including anodes [7–9]. Due to this unique property, *Shewanella* cells can be electrochemically cultured without the additional requirement for toxic mediator compounds, which are typically required for the electrochemical cultivation of living cells [7–9].

**Figure 1:**
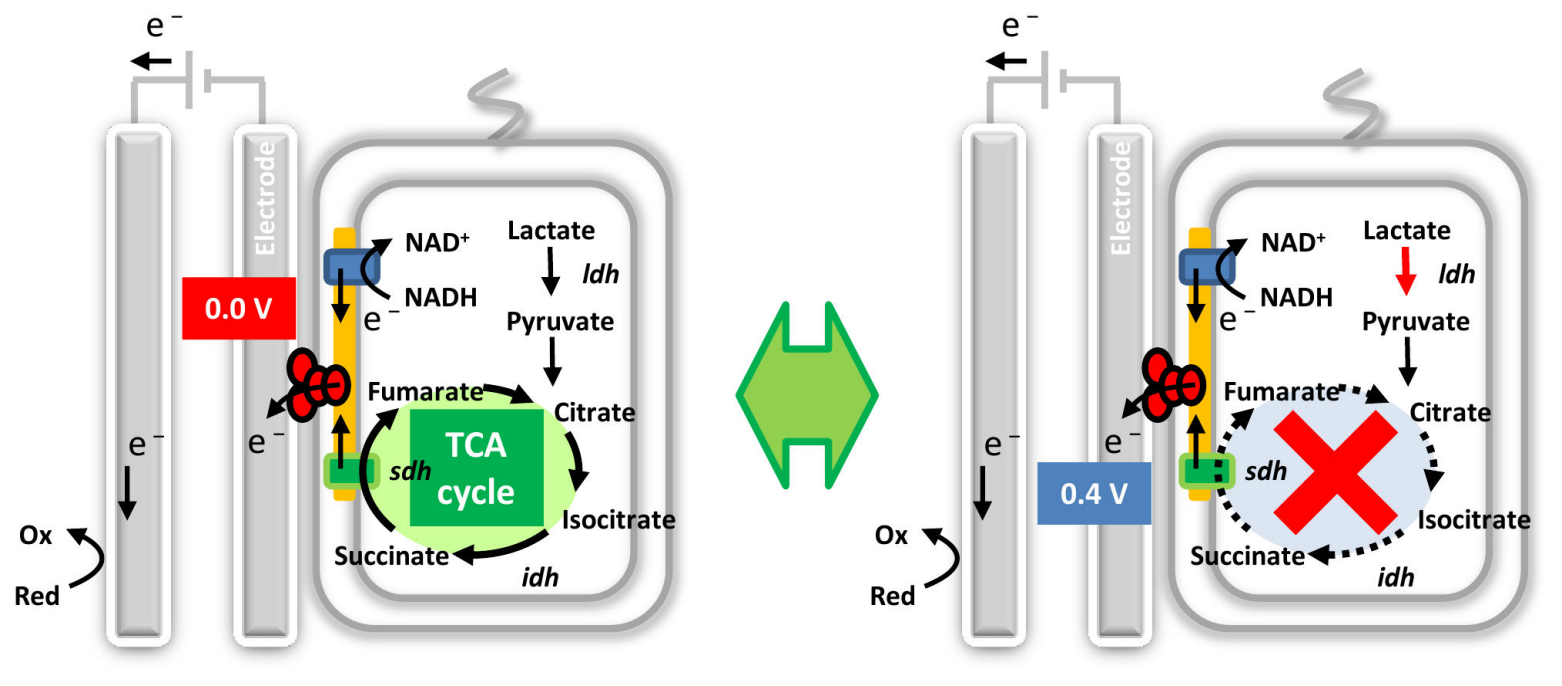
Schematic illustration of electrochemical switching in the TCA metabolic pathway of *Shewanella* cells. Left, Activation of TCA cycle in cells grown at 0.0 V. Right, Deactivation of TCA cycle in cells grown at 0.4 V.

In *Shewanella* cells, respiratory electrons are transferred to an external electrochemical circuit via the OMCs (Figure 1). In an electrochemical cell, the anode potential can be precisely controlled and the flow rate of the extracellular electron transfer reactions can be detected as an electric current; namely, the relationship between the energy level of the acceptor (i.e., the anode) and respiration activity can be quantitatively investigated by voltammetry. Thus, we can quantitatively obtain the redox-dependency of the intracellular electric current in living whole-cells. Besides, in this case, we can also expect that the intracellular redox balance is influenced by the operation of the anode potential through the electric conduit of OMCs, resulting in the change of metabolic property. Herein, we report that the TCA cycle in the bacterium *Shewanella* is reversibly gated by electrochemical technique.

## Results and Discussion

**Figure 2:**
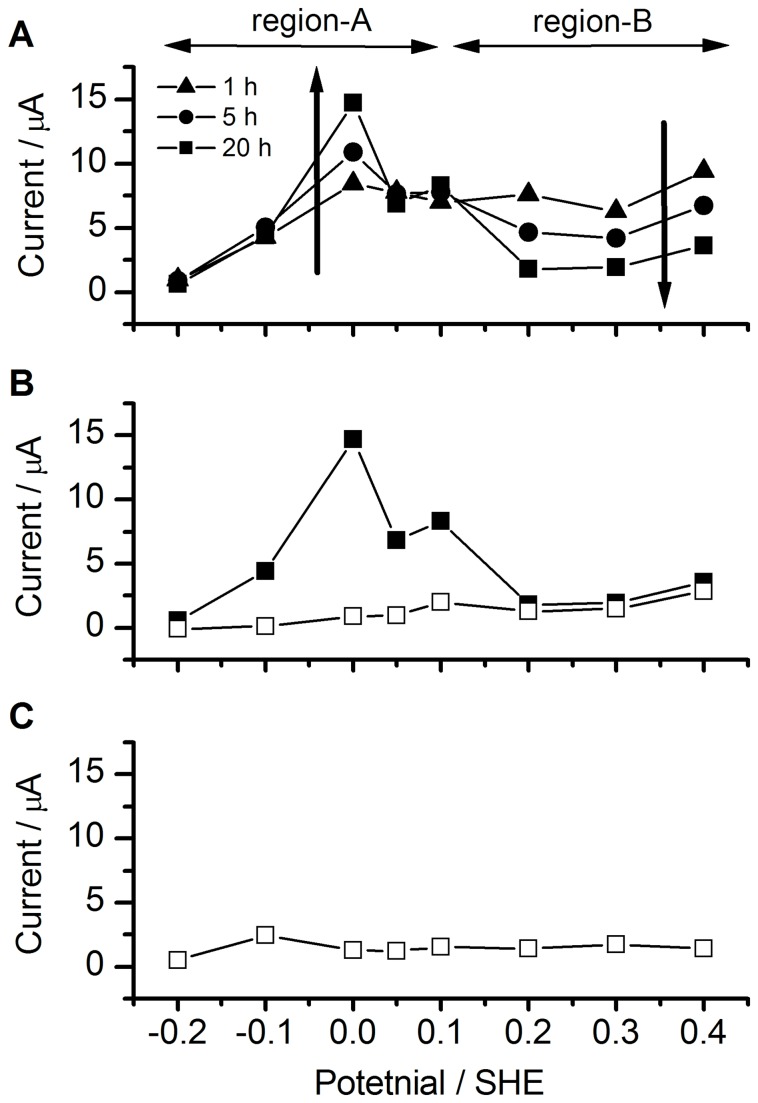
Potential dependency of the microbial current. (A) Microbial current by wild-type cells after 1 h (triangles), 5 h (circles), and 20 h (squares) of electrochemical cultivation at the indicated potentials. (B) Microbial current by wild-type cells just before (black squares) and after (white squares) the addition of malonic acid into the electrochemical system. (C) Microbial current by *sdh* deficient mutant cells after 20 h of electrochemical cultivation.

The respiration activity of *Shewanella* cells was first evaluated by measuring current generation after 1 h (triangles), 5 h (circles), and 20 h (squares) of electrochemical cultivation at various poised potentials (Figure 2A). It can be seen that the current continued to increase in region A (-0.2 ~ 0.1 V vs. SHE), whereas it became lower with time in region B (0.1 ~ 0.4 V vs. SHE). It is now known that such potential dependent behavior was observed when the OMCs can possess direct electrical interaction with the electrode substrate [10,11]. Essentially same potential dependency of the microbial current was generally observed even when other material such as carbon and gold was used as the electrode substrate. At all examined potentials, in-situ electron microscopic inspection of the electrode surface did not reveal any obvious differences in the cell density on the electrode surface. In addition, the current instantaneously responded against the alternate change of the applied potential in a reversible manner, indicating that the cytotoxic effect by the long-time electrochemical cultivation is limited for the present case (Figure S1). These results indicate that the change of microbial current in response to the potential was not a reflection of the number of cells contributing to the current generation, but rather of the metabolic activity of cells. Note that the observed potential dependency of the microbial current is opposed to the response derived from thermodynamics.

**Figure 3:**
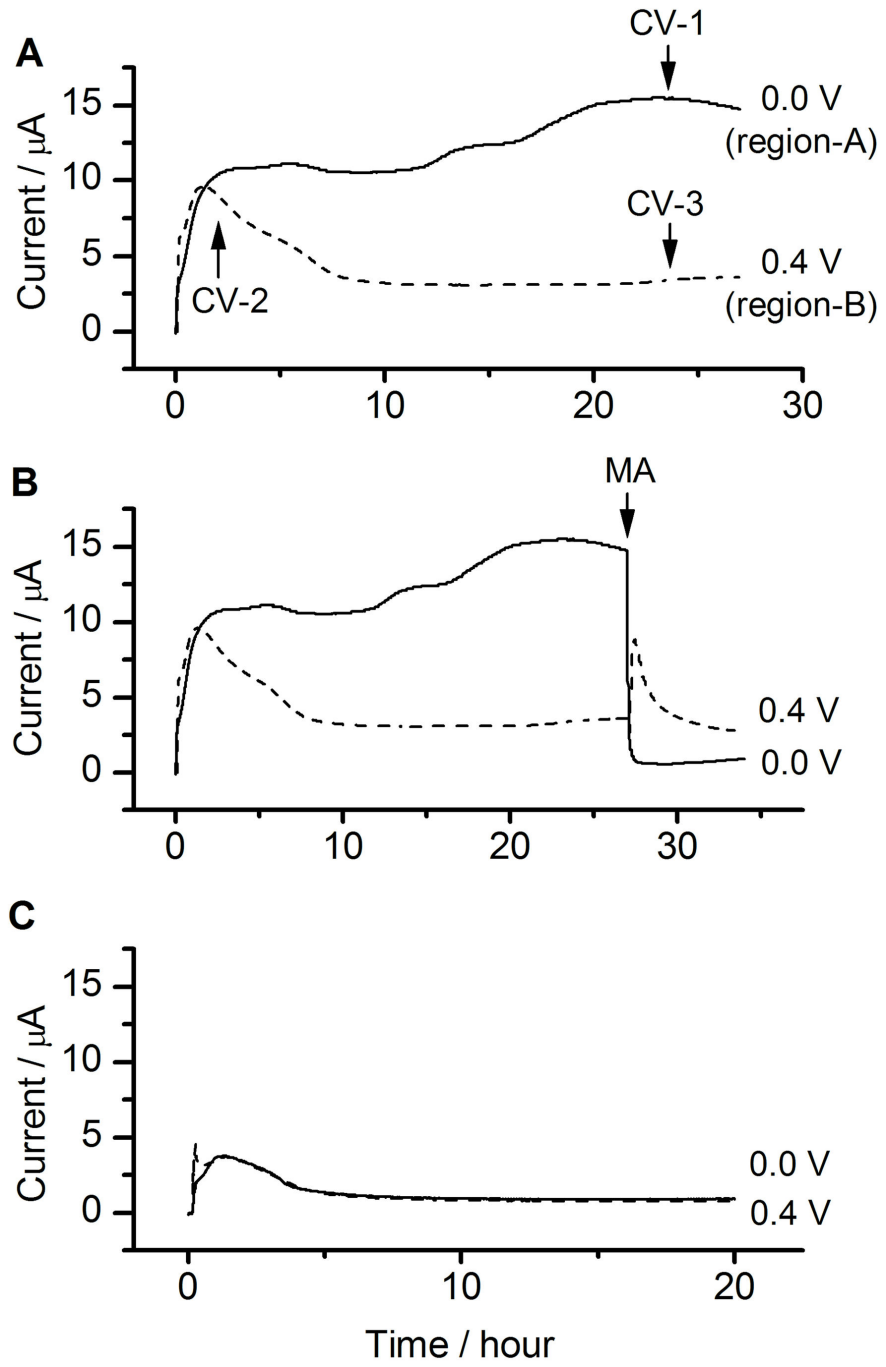
Time courses of the microbial current. (A) Time courses of microbial current by wild-type cells at 0.0 V (solid curve) and 0.4 V (dashed curve). (B) Time courses of microbial current by wild-type cells before and after the addition of malonic acid (MA; indicated by the arrow at 0.0 V (solid curve) or 0.4 V (dashed curvee). (C) Time courses of microbial current by the *sdh* deficient mutant cells at 0.0 V (solid curve) or 0.4 V (dotted curve).

Based on the above results, it was estimated that the highest current values in regions A and B differed by approximately ten fold. We speculate that this large difference in current was caused only by activation/deactivation of the TCA cycle. Metabolic product analysis is the general method for investigation of the metabolic pathway. However, it is technically difficult to investigate the microbe, which located just on the electrode surface. Instead, we investigate the TCA cycle activity by observing the metabolic current response against the addition of TCA cycle inhibitor compounds. Thus, the potential-dependent activity of the TCA cycle in cells was examined on-line only by adding malonic acid, a representative inhibitor of the TCA cycle [12], into the electrochemical system and then observing the degree of the current reduction at various potentials. At a poised potential of 0.0 V, the microbial current instantaneously and drastically decreased upon the addition of malonic acid (Figure 3A, solid curve), whereas no decrease in the current was observed at 0.4 V (Figure 3A, dashed curve). To investigate the details of the potential dependent activity of the TCA cycle, the microbial current just before (circles) and after (squares) the addition of malonic acid into the electrochemical system is plotted against the poised potential (Figure 2B). It can be clearly seen that current suppression by malonic acid occurred only in the region of higher current (region A), indicating that the TCA cycle is only active in cells in region A. Note that small current observed even after the addition of inhibitor was expected to be through the glycolysis pathway. Namely, the microbes produce NADH through the glycolysis pathway even after the addition of inhibitor. Then, the electrons in NADH are finally transferred to the anode via the flavin-OMC complexes, generating the electric current. A similar trend was also found using other inhibitor compounds of the TCA cycle (Figure S2). The potential-dependent activity of the TCA cycle was further confirmed by experiments performed with *sdh*-deficient mutant cells, which lack complex II and are therefore expected to have a completely impaired TCA cycle. The electrode-potential dependence of the microbial current observed for the wild-type cells was not detected in the *sdh*-deficient mutant cells (Figures 2C, 3C). These results further support the finding that the current observed in region A is due to the activity of the TCA cycle in wild-type cells. Although *Shewanella* does not maintain significant ﬂux through the TCA cycle under anaerobic conditions [13,14], our present results demonstrate that the TCA cycle in *Shewanella* cells functions at potential region A under electrochemical culture conditions.

**Figure 4:**
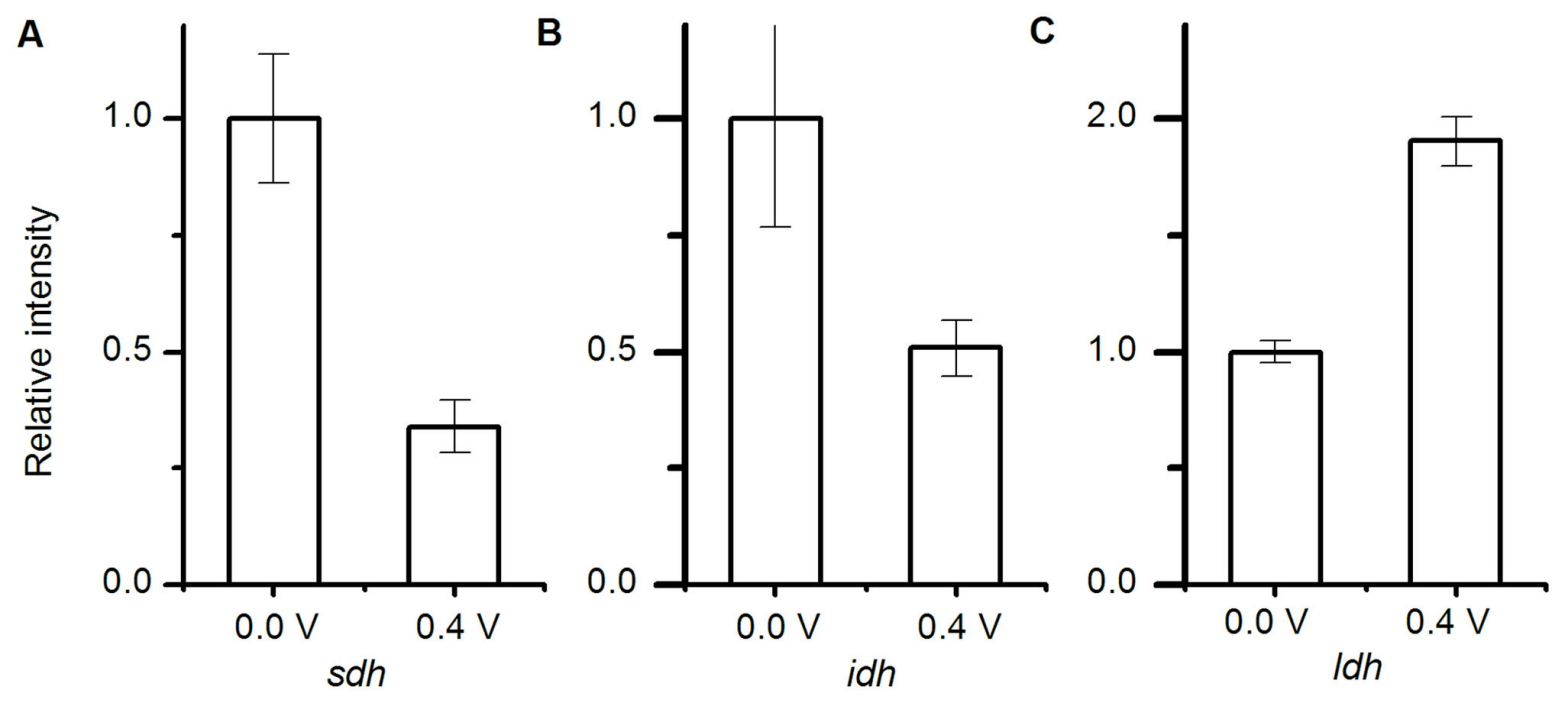
Electrode potential dependence of gene expression profiles. Quantitative RT-PCR analyses of genes encoding (A) succinate dehydrogenase, (B) isocitrate dehydrogenase, and (C) lactate dehydrogenase in wild-type cells cultured electrochemically at 0.0 V or 0.4 V. Error bars represent SD (n=3).

To investigate and compare the gene expression profiles of genes related to the TCA cycle in cells cultured at 0.0 V (region A) and 0.4 V (region B), quantitative reverse transcription PCR (qRT-PCR) analyses was performed. In this analysis, we focused on the genes *sdh* (Complex II) and *idh*, encoding isocitrate dehydrogenase, which catalyzes the rate-limiting reaction in the TCA cycle. The qRT-PCR analyses revealed that both genes were up-regulated over two fold in cells grown at 0.0 V (region A) compared with cells grown at 0.4 V (region B) (Figure 4A, 4B). We also evaluated the expression level of the ldh (lactate dehydrogenase) gene, which is related to glycolysis, and found that in contrast to the *sdh* and *idh* genes, expression of the *ldh* gene was higher in cells grown at 0.4 V than at 0.0 V (Figure 4C). The fact that the *sdh* (and *idh*) and *ldh* genes exhibited opposite potential dependency is important as it excluded the possibility that just the whole-cell metabolic activity depends on the anode potential. Taken all together, our results indicate that the TCA-cycle in *Shewanella* cells was electrochemically gated in a potential-dependent and reversible manner.

**Figure 5:**
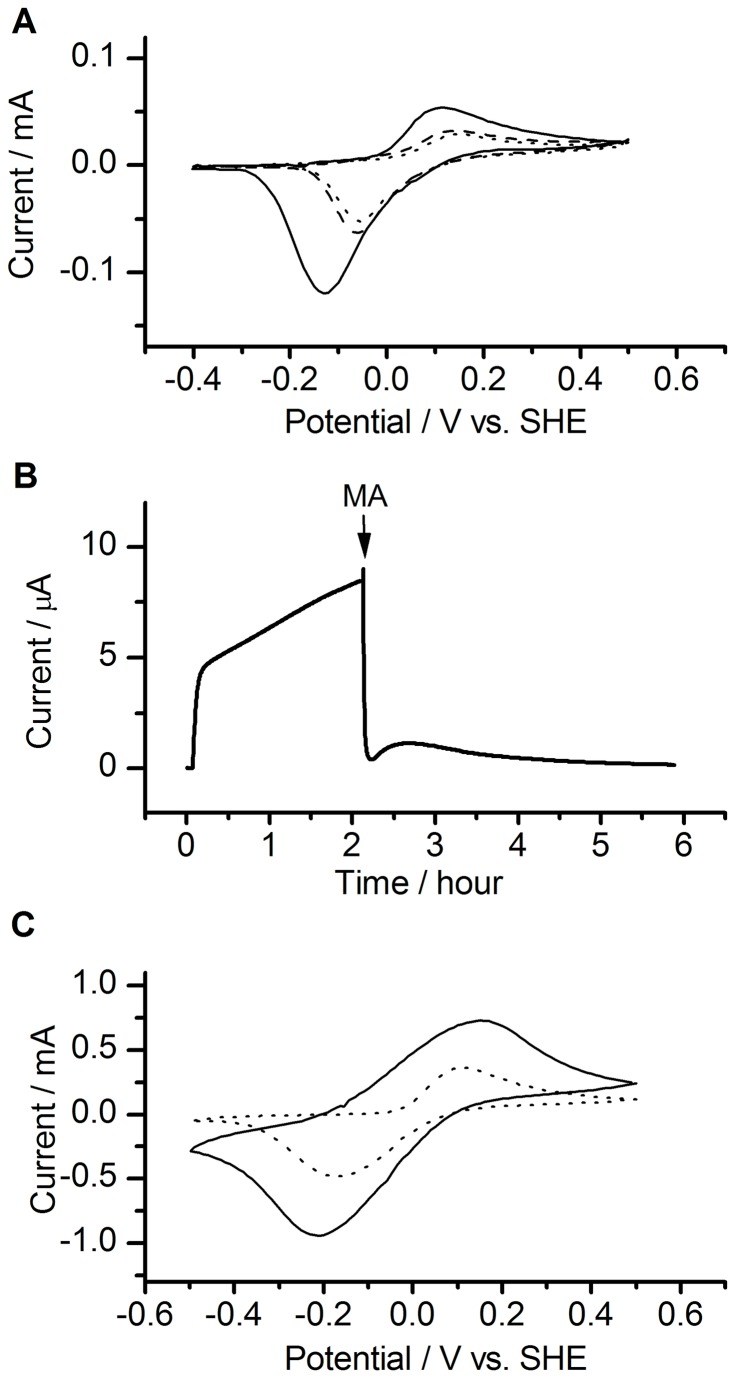
Relationship between TCA cycle activity and cyclic voltammogram (CV) peak area. (A) Whole-cell CV in wild-type cells at point 1 (dotted curve), point 2 (dashed curve), and point 3 (solid curve). The scan rate was 50 mV/s. (B) Time course of microbial current in wild-type cells at 0.4 V. Malonic acid (MA) was injected into the electrochemical system at the time point indicated by the arrow. (C) Whole-cell CV in the *sdh* deficient mutant cells cultured electrochemically at 0.0 V (dotted curve) or 0.4 V (solid curve). The scan rate was 50 mV/s.

As described above, the TCA cycle is activated and deactivated in regions A and B, respectively. What triggers the electrochemical gating of the TCA cycle? This question can be solved by considering the degree of electrical interaction between microbial cells and the anode. The degree of the electrical interaction can be estimated from the intensity of the peak assignable to flavin-OMC complexes in cyclic voltammograms (CV) [15]. Note that the difference in the CV peak intensity was not caused by the difference in the number of bacterial cells on the electrode, but by the difference in the number of flavin-OMC complex that are electrically interacting with the electrode [16]. The CVs obtained at points 1 (region A), 2 (early stage in region B), and 3 (later stage in region B) in Figure 3A are shown in Figure 5A. As have been reported previously, a clear correlation between the CV peak intensity and the microbial current was confirmed [10,11]. Namely, the CV peak intensity was low when the microbial current was high (i.e., at points 1 and 2), and it was high when the microbial current was low (i.e., at point 3). In a good harmony with this fact, the microbial current at the early stage in region B (i.e., at the point 2) decreased when malonic acid is added to the system (Figure 5B). Notably, the potential-dependent behavior of the intensity of the CV peak was conserved even in *sdh*-deficient mutant cells (Figure 5), although the potential dependency of the TCA cycle activity was lost in the mutant cells (Figures 2C, 3C). This fact indicates that the TCA-activity does not determine the intensity of the CV peak (i.e., the degree of electrical connection), but the electrical connection between OMCs and the anode affects on the TCA-cycle activity. Taken all together, we can conclude that the TCA cycle is deactivated when the microbial cells are highly electrically connected to the anode.

In such conditions that the microbial cells are highly electrically connected to the electrode (i.e., when the CV peak intensity is large), the intracellular redox atmosphere is expected to be oxidative due to the efficient extracellular transfer of electrons via oxidized flavin-OMC complexes. Sucheta et al. [17–20] revealed by electrochemical technique that complex II, which is located in the inner membrane and is an important component of the TCA cycle, is deactivated in its oxidative condition. Thus, it appears that this response is an inherent safety mechanism against oxidative stress. Such unique enzymatic characteristics may also participate in the gating of TCA cycle in *Shewanella* . As we mentioned, the intracellular redox conditions can be directly regulated by the operation of the electrode potential through the electric conduit constructed by flavin-OMC complexes in *Shewanella* . Studies for clarifying the biomolecular mechanism of redox dependent regulation in TCA cycle are currently in progress in our laboratory.

## Conclusions

In conclusion, we demonstrate that TCA cycle is electrochemically gated in bacterium *Shewanella* . Recently, the importance of the controllability of intracellular electron transfer pathway and the redox atmosphere has been recognized in the field of bio-production. We anticipate that our findings will contribute to a deeper understanding of redox-dependent regulation systems in living cells, in which the intracellular redox atmosphere is a critical factor determining the regulation of various metabolic and genetic processes.

## Materials and Methods

### Microbe preparation

*Shewanella* *loihica* PV-4 (wild type and *sdh* deficient mutants) and *Shewanella oneidensis* MR-1 were cultured aerobically in 10 mL marine broth (MB) (20 g/L) at 30 ^°^C for 24 h. Cells were collected by centrifugation, washed three times with defined medium (DM; 2.5 g NaHCO_3_, 0.08 g CaCl_2_·2H_2_O, 1.0 g NH_4_Cl, 0.2 g MgCl_2_·6H_2_O, 10 g NaCl, and 7.2 g HEPES per liter, pH 7.8) and then resuspended in 4 mL DM supplemented with 10 mM lactate as a carbon source for further cultivation at 30 °C for 24 h. The concentration of the cell suspension in the electrochemical cell was set to be the optical density at 600 nm of 2.0.

### Gene disruption

The in-frame disruption of the *sdhA* and *sdhB* gene in strain PV-4 was performed using suicide plasmid pSMV-10 and a two-step homologous recombination method as described previously [21]. Brieﬂy, a 1.5-kb fusion product, consisting of an upstream sequence of the target gene (approximately 700 bp), replacement sequence (approximately 18 bp), and downstream sequence (approximately 700 bp), was constructed by PCR and in vitro extension using primers (Table S1). This fusion product was ligated into pSMV10 at the SpeI site. The resultant plasmid was introduced into PV-4 by ﬁlter mating with *E. coli* WM6026. Transconjugants were selected on LB plates containing Km (kanamycin), and these single-crossover clones were further cultivated for 20 h in LB medium lacking the antibiotics. The cultures were then spread onto LB plates containing 10% (wt/vol) sucrose to isolate Km-sensitive double-crossover mutants. The disruption of the gene in these strains was conﬁrmed by PCR. The broad-host-range plasmid pBBR1-MCS524 was used to generate a complementation vector, to which a DNA fragment ampliﬁed from the genome of PV-4. The complementation vectors were introduced into *Shewanella* mutant strains with the assistance of *E. coli* WM6026, and transconjugants were selected on LB agar plates containing kanamycin.

### Electrochemical characterization

A single-chamber (4 mL in volume), three-electrode system was used to monitor the electrochemical behavior of microbes. Ag/AgCl (sat. KCl) and platinum wire were used as the reference and counter electrodes, respectively, and ITO-coated glass was used as the working electrode and was mounted on the bottom of the reactor. Prior to the injection of microbial cells, the electrode potential was poised at a potential in the range between -0.2 and 0.4 V vs. SHE. Then, microbial cells are injected into the reactor, which gradually settled on the electrode surface with time, resulting in formation of biofilms on the anode associated with microbial current generation. DM containing lactate (10 mM, pH 7.8) was used as the electrolyte and was deaerated by N_2_ bubbling for 30 min before measurements. The temperature of the system was maintained at 30 °C. A potentiostat (Hokuto Denko Co. Ltd., HZ-5000) was used for all the electrochemical measurements in this study.

### Quantitative RT-PCR analysis

Total RNA from *Shewanella* cells was extracted with Trizol reagent (Invitrogen) and purified with the RNeasy Mini Kit and RNase-Free DNase Set (Qiagen) according to the manufacturer’s instructions. Reverse transcription (RT) and subsequent quantitative PCR were carried out with a LightCycler 1.5 instrument (Roche) according to the manufacturer’s instructions. The PCR mixture (20 µL) contained 15 ng RNA, 1.3 µL of 50 mM Mn(OAc) 2 solution, 7.5 µL LightCycler RNA Master SYBR Green I (Roche), and 0.15 µM of the primer sets as follow (Supporting Information, Table S2). For the standard curves, DNA fragments of the target genes (*sdh*, *idh*, *ldh*, and 16s rDNA) were amplified by PCR with total DNA from strain PV-4 and the primer sets for the respective target genes (Table S2), and a dilution series of the template cDNA was subjected to quantitative PCR analyses. The specificity of the QT-PCR was verified by dissociation curve analysis. The mRNA levels of the target genes (*sdh*, *idh*, and *ldh*) were normalized to that of the reference gene (16s rDNA).

## Supporting Information

Figure S1 **Time course of microbial current obtained when the applied potential was alternated between 0.0 V and 0.4 V.**
(TIF)

Figure S2 **Effect of TCA cycle inhibitor compounds for current generation is *Shewanella* cells.** Time course of microbial current before and after the addition of (A) carboxin and (B) 2-thenoyltrifluoroacetone (TTF) at 0.0 V or 0.4 V, respectively.
(TIF)

Table S1 **Oligonucleotide sequences of the primers used to construct the *S* *. loihica* PV-4 in-frame deletion mutants.**
(DOC)

Table S2 **The primers used for Quantitative RT-PCR analysis.**
(DOC)
