# Supplementary material for: Electrochemical Gating of Tricarboxylic Acid Cycle in Electricity-Producing Bacterial Cells of Shewanella
Source: PLoS One. 2013 Aug 20;8(8):e72901. doi: 10.1371/journal.pone.0072901 (PMC3748093; doi:10.1371/journal.pone.0072901)
Supplement: Table S1 — Oligonucleotide sequences of the primers used to construct the S . loihica PV-4 in-frame deletion mutants. (DOC) [file pone.0072901.s003.doc]

| **Primer** | **Sequence** |
| --- | --- |
| sdhA_5-O-SpeI | CATCACTAGTCAATGGAGCAGAGTGAGCA |
| sdhA_5-I | GGTCACACCAGCACCTGAGACTGCATCAAACRCACG |
| sdhB_3-O-SpeI | TAGCACTAGTGCCGATAGCAGCACAATAGG |
| sdhB_3-I | TCAGGRGCTGGTGTGACCATGCTGCTTAAGCGGAGCT |
