# Supplementary material for: Electrochemical Gating of Tricarboxylic Acid Cycle in Electricity-Producing Bacterial Cells of Shewanella
Source: PLoS One. 2013 Aug 20;8(8):e72901. doi: 10.1371/journal.pone.0072901 (PMC3748093; doi:10.1371/journal.pone.0072901)
Supplement: Table S2 — The primers used for Quantitative RT-PCR analysis. (DOC) [file pone.0072901.s004.doc]

| **Primer** | **Sequence(5'-3')** |
| --- | --- |
| sdh-f | CGTGAGTTTGATGCAGTCGT |
| sdh-r | GTGCTGTTCCCAATGGTCTT |
| idh-f | GGCACGTTATGACAACATCG |
| idh-r | GCTCGTAGGCGAAGGTAGTG |
| ldh-f | ATGGTTTCGAGGTGATCTGC |
| ldh-r | ATCAGGGCCACAGTATGCTC |
| 16sr-f | GGACGGGTGAGTAATGCCTA |
| 16sr-r | ACATCCAATCGCAGAAGGTC |
